# Controlling the causative agents of coccidiosis in domestic chickens; an eye on the past and considerations for the future

**DOI:** 10.1186/s43170-021-00056-5

**Published:** 2021-09-27

**Authors:** Elizabeth Attree, Gonzalo Sanchez-Arsuaga, Michelle Jones, Dong Xia, Virginia Marugan-Hernandez, Damer Blake, Fiona Tomley

**Affiliations:** 1 Department of Pathobiology and Population Sciences, The Royal Veterinary College, North Mymms, Hertfordshire, United Kingdom; 2 Department of Clinical Science and Services, The Royal Veterinary College, North Mymms, Hertfordshire, United Kingdom; 3 UKRI GCRF One Health Poultry Hub, Ahmedabad, India

## Abstract

Coccidiosis is a potentially severe enteritis caused by species of obligate intracellular parasites of the genus *Eimeria.* These parasites cause significant economic losses to the poultry industry, predominantly due to compromised efficiency of production as well as the cost of control. These losses were recently estimated to cost chicken producers approximately £10.4 billion worldwide annually. High levels of *Eimeria* infection cause clinical coccidiosis which is a significant threat to poultry welfare, and a pre-disposing contributory factor for necrotic enteritis. Control of *Eimeria* parasites and coccidiosis is therefore an important endeavour; multiple approaches have been developed and these are often deployed together. This review summarises current trends in strategies for control of *Eimeria*, focusing on three main areas: good husbandry, chemoprophylaxis and vaccination. There is currently no “perfect solution” and there are advantages and limitations to all existing methods. Therefore, the aim of this review is to present current control strategies and suggest how these may develop in the future.

## Introduction

Coccidiosis is an enteric disease caused by obligate intracellular protozoa of the genus *Eimeria,* highly host-specific apicomplexan parasites closely related to the causative agents of many other human and animal diseases including species of: *Babesia*, *Besnoitia*, *Cryptosporidium*, *Cystoisospora*, *Neospora*, *Plasmodium*, *Sarcocystis*, *Theileria*, and *Toxoplasma.* Seven species of *Eimeria* that infect domestic chickens (*Gallus gallus domesticus*) (Reid et al. 2014) are recognised as globally ubiquitous (*E. acervulina, E. brunetti, E. maxima, E. mitis, E. necatrix, E. praecox* and *E. tenella*). Additionally, three cryptic *Eimeria* operational taxonomic units (OTUs) have been detected in chickens from across several continents (Cantacessi et al. 2008; Clark et al. 2016; Hinsu et al. 2018; Hauck et al. 2019), and on the basis of genotypic and phenotypic properties these were recently proposed to be previously unrecognised parasite species and given the names *Eimeria lata*, *Eimeria nagambie* and *Eimeria zaria* (Blake et al. 2021).

All species of *Eimeria* that infect chickens can cause coccidiosis, but four of these (*E. acervulina, E. maxima, E. necatrix* and *E. tenella*) are generally considered most important due to their pathogenicity, global prevalence and overall economic impact. The emergence of what appear to be previously undetected *Eimeria* species suggests that there is potential for additional pathogenic and economic threats in the future. All three of the newly described species have negative impacts on chicken production parameters and the live vaccines currently available to control coccidiosis confer very low or no protection against them, most likely because of the species-specific nature of immune protection induced by *Eimeria* infection (Fornace et al. 2013; Blake et al. 2021).

*Eimeria* infection of chickens is initiated by ingestion of sporulated oocysts from the environment (e.g., faeces and contaminated litter) leading to invasion of epithelial cells lining the intestinal tract by released sporozoites. Each *Eimeria* species exhibits marked tropism for specific regions of the gut, (see Table 1; Lai et al. 2011). The lifecycle of wild-type *Eimeria* species in chickens comprises a stable number of rounds of asexual reproduction (schizogony), typically three or four depending on species (McDonald and Rose 1987; Walker et al. 2013), with new enterocytes invaded for each round, before a sexual phase termed gametogony ensures. Following fertilisation, progeny oocysts are excreted and these sporulate in the external environment, becoming infectious to new hosts. The pathology associated with each *Eimeria* species varies, with infection occurring in different sections of the intestine and causing either malabsorptive (*E. acervulina*, *E. maxima*, *E. mitis* and *E. praecox*) or haemorrhagic (*E. brunetti*, *E. necatrix* and *E. tenella*) disease, Table 1 (Williams 1998; Blake and Tomley 2014; Burrell et al. 2020).

**Table 1:** Pathogenicity, type of disease caused and region of development of the seven recognised species of *Eimeria* that cause coccidiosis in chickens (Blake and Tomley 2014; Cisman et al. 2020; Horton-Smith and Long 1965; Joyner 1958; Joyner and Davies 1960; Long, 1967; 1968; Reid et al. 2014; Williams 1998)

*Eimeria* Species	Gross pathological lesions	Haemorrhagic disease	Malabsorptive disease	Region of development
*E. brunetti*	✓	✓	✕	Lower intestine
*E. necatrix*	✓	✓	✕	Mid-intestine and caeca
*E. tenella*	✓	✓	✕	Caeca
*E. acervulina*	✓	✕	✓	Duodenum
*E. maxima*	✓	✕	✓	Mid-intestine
*E. mitis*	✕	✕	✓	Mid-intestine
*E. praecox*	✕	✕	✓	Duodenum

Coccidiosis varies significantly in its severity and impact on individual chicken health and flock productivity. Depending on parasite species, infectious dose, age and immune status of the host, infected chickens may show few, if any, clinical signs or can suffer effects ranging from reduction in expected weight gain, feed conversion or egg-production, failure to thrive due to malabsorption or diarrhoea, to severe enteritis and death. At flock level, an important consideration is the overall economic burden imposed by coccidiosis and the ongoing cost of its control. In 1995, the global cost was estimated at  ~ £38 million annually, with 98% of that cost attributed to broilers (Williams 1999; Kadykalo et al. 2018). Today that figure has been recalculated as  ~ £10.4 billion annually, taking account of current global poultry production and disease prevalence (Blake et al. 2020). The vast increase in cost is likely multifactorial and includes massive expansion of the industry in the past 25 years, increased broiler growth rates resulting in reduced growing periods (from  ~ 45 days in 1995 to  ~ 31–37 days today for intensive systems (Williams 1999; Kadykalo et al. 2018), and hence less time for birds to develop immunity and recover losses in weight gain.

Due to the significant economic and animal welfare impacts of coccidiosis, the need for ongoing management and control of *Eimeria* parasites remains essential. Management and control strategies can be broadly categorised into three main areas: animal husbandry, chemoprophylaxis and vaccination. In this review, the development, advantages and limitations of each approach will be discussed, together with a summary of the alternative strategies available.

## Husbandry

Good husbandry is essential for effective control of clinical and subclinical coccidiosis. Key factors include consideration of the flock environment, such as litter quality, ventilation rate and humidity, as well as stocking density (Long et al. 1976; Bumstead and Millard 1992; Kim et al. 2008; Williams et al. 2009; Bacciu et al. 2014; Blake et al. 2005, 2015). In a broader context, the impact of host genetics can be beneficial, choosing lines or selectively breeding for individuals that are more resistant to *Eimeria* and the consequences of coccidiosis (Palafox et al. 1949; Champion 1954; Rosenberg et al. 1954; Jeffers et al. 1970; Swaggerty et al. 2011; Boulton et al. 2018a,b).

### *Impact of *climatic* factors*

*Eimeria* oocysts have a tough multi-layered wall rendering them relatively resistant to most disinfectants. However, high temperatures (>  50 °C) and ammonia can disrupt oocyst integrity (Fish 1931; Horton-Smith et al. 1940; Williams 1997; Allen and Fetterer 2002). Humidity levels in the immediate environment affect the rate and efficiency of oocyst sporulation as well as subsequent longevity. Damp conditions in poultry houses can be advantageous for *Eimeria* survival, with examples such as water spillages or high rainfall resulting in humidity in excess of 60% (Anderson et al. 1976; Khan et al. 2006; Nematollahi et al. 2009; Awais et al. 2012). Open-house poultry rearing is practiced in many tropical and subtropical areas and is common in backyard production systems. In these external environments, under optimal conditions (25–30 °C,  ~ 75% humidity with aeration), sporulated oocysts can survive for up to 602 days (Farr and Wehr 1949; Edgar 1955; Graat et al. 1994; Waldenstedt et al. 2001; Fatoba and Adeleke 2018). Under drier conditions and lower temperatures sporulation has been observed to be delayed (Musa et al. 2010).

Ambient temperatures of  ~ 25 °C favour *Eimeria* oocyst sporulation; however, oocysts can survive temperatures as low as 4 °C (Anderson et al. 1976; Fayer 1980). In tropical settings it has been reported that oocyst sporulation and survival is favoured during and directly following rainy seasons with higher prevalence of *Eimeria* infection observed, for example in: Egypt during winter (rainy season December-February), Ethiopia after the rainy season in October, and the Kashmir valley, India, between September and November (Oikawa et al. 1979; Dar and Anwar 1981; Khan et al. 2006; Haug et al. 2008; Al-Gawad et al. 2012; Awais et al. 2012; Luu et al. 2013; Ahad et al. 2015; Sharma et al. 2015). However, higher temperatures are inhibitory, limiting replication. For example, the highest prevalence of coccidiosis in Pakistan was detected towards the end of the monsoon season as the ambient temperature decreased to  ~ 25 °C (Awais et al. 2012), in common with previous studies of ambient temperature and season (Anderson et al. 1976; Dar and Anwar 1981; Khan et al. 2006).

While it appears that oocyst sporulation and survival is favoured in environments with higher humidity levels, especially following the main rainy seasons, it is not possible to solely ascribe high or low *Eimeria* prevalence exclusively to climatic factors. A lack of awareness of transmission and control; and limited resources are also key factors. These are often observed in the poultry management practices of low and middle income countries (Williams 1998; Lawal et al. 2016).

### *Poultry *housing

In poultry houses, *Eimeria* oocysts can accumulate in the litter, feeders and drinkers (Gross 1985; Khan et al. 2006). Where there is high stocking density, the faecal-oral transmission of sporulated oocysts may increase rapidly within a short period of time (Williams 1995; Trees et al. 2001). Reducing *Eimeria* infection of chickens can be achieved by limiting oocyst sporulation in the environment, primarily by maintaining dry litter and improving ventilation to a poultry house (Stayer et al. 1995; Etuk et al. 2004). It has also been observed that oocyst viability starts to decline in broiler house litter after approximately 3 weeks, likely due to high environmental ammonia levels (Horton-Smith et al. 1940; Williams 1995). Minimising exposure to common stressors including overcrowding, high temperatures, debeaking, restriction of feed and dietary deficiencies can strengthen chicken immune responses to *Eimeria* (Williams 1998). Coccidiosis control may also be achieved through reducing water, wind, invertebrate, vermin and other mechanical dispersal of *Eimeria* oocysts by litter changes (Fayer 1980).

### *Chicken breed *resistance* and susceptibility*

It has long been recognised that different chicken breeds can exhibit varied levels of “susceptibility” or “resistance” to *Eimeria* species, including tolerance to infection and rate of recovery from the pathological consequences of infection (Palafox et al. 1949; Champion 1954; Rosenberg et al. 1954; Jeffers et al. 1970; Bishop and Woolliams 2014; Boulton et al. 2018a). Differences in susceptibility to *Eimeria* infection have been found between and within outbred and inbred chicken breeds/lines, with reports of more than two-fold variations in overall susceptibility to *Eimeria* species (Bumstead and Millard 1992; Zhu et al. 2003; Pinard-van der Laan et al. 2009) for example, differences in response to *E. tenella* challenge have been identified between the relatively resistant Egyptian Fayoumi and more susceptible White Leghorn breeding lines (Pinard-van der Laan et al. 1998, 2009). Variation in phenotypes such as pathology and body weight gain during *Eimeria* infection has been used in genetic mapping studies to identify quantitative trait loci (QTL) regions associated with *E. maxima* and *E. tenella* resistance in chickens (Pinard-van der Laan et al. 2009; Bacciu et al. 2014; Hamzić et al. 2015). It is however notable that an inverse relationship has been observed between susceptibility to *E. tenella* and susceptibility to other *Eimeria* species such as *E. maxima* (Bumstead and Millard 1992), possibly limiting opportunities for breed improvement. Nonetheless, improved understanding of the genetic basis of coccidiosis resistance/tolerance/susceptibility traits through identification of genetic markers could be used to influence chicken breeding decisions, aiding future control of coccidiosis (Pinard-Van Der Laan et al. 1998; Boulton et al. 2018a). Genetic selection is a long-term approach that must be implemented throughout generations of chickens (Hamzić et al. 2015). However, in the long term it might prove more cost-effective, with fewer host and environmental effects, than chemoprophylactic and vaccinal methods of control.

## Chemoprophylaxis

Control of coccidiosis by chemical prophylaxis has been practised in poultry production since 1948 (Grumbles et al. 1948; Chapman 2009; Kadykalo et al. 2018). Chemical intervention includes the use of two categories of anticoccidial compound: organic or synthetic. Organic compounds used for coccidiosis control are typically produced from fermentation reactions whereas synthetic compounds arise from chemical synthesis (Osweiler 2011; Noack et al. 2019). Ionophores, named due to their ion bearing properties, are a group of organic compounds that bind and transport ions across biological membranes, the majority of those used for control of coccidiosis are produced from fermentation reactions by *Streptomyces* species (Berger et al. 1951; Ryley and Wilson 1975; Remnant 2007; Osweiler 2011; Dorne et al. 2013; Clarke et al. 2014; Noack et al. 2019).

From a regulatory perspective, there is an important difference in the classification of anticoccidial drugs across different regions of the globe. For example, in Europe ionophores are classified as feed additives, whereas in the USA they are instead classified as polyether ionophorous antibiotics (Chapman 2001, 2005). It is important to note that compounds not classified as feed additives can still be administered in feed. Anticoccidial drugs in feed have been regulated in the EU since the 1970’s (Hafez 2008), with 11 compounds currently licenced in the EU as feed additives, detailed in Table 2 (Goetting et al. 2011; Peek and Landman 2011; Dorne et al. 2013; EU 2021). Of these, some are also licenced for use in other production systems such as ruminants (lasalocid, monensin, halofuginone, diclazuril and decoquinate), pigs (semduramicin and narasin), turkeys (lasalocid, monensin, diclazuril, halofuginone, narasin, nicarbazin and salinomycin) and rabbits (diclazuril) (Mooney et al. 2020; NOAH 2021). Some other compounds are licenced for therapeutic interventions against coccidiosis, including toltrazuril, but these are classified as pharmaceuticals.

**Table 2:** The 11 anticoccidial compounds authorised as feed additives in the European Union (Abbas et al. 2011a; Anadón and Martínez-Larrañaga 2014; AnimalDrugs@FDA 2021; Belanger et al. 2013; Bozkurt et al. 2013; Castanon 2007; Chapman 1997; Clarke et al. 2014; Dorne et al. 2013; Dubey 2019; EU 2021; Gerhold 2014; Goetting et al. 2011; Hafez 2008; Harder and Haberkorn 1989; Kant et al. 2013; Noack et al. 2019; NOAH 2021; Salisch 1989)

Compound	Category	Effective Conc. ppm	Mode of action	Example products and Withdrawal period (days)^**a**^	Year of introduction
Lasalocid	Divalent ionophore	75–125	Disruption of ion balance across biological membranes and therefore membrane potential, stimulation of mitochondrial ATPase activity	Avatec—5	1977
Maduramicin	Monovalent glycosidic ionophore	5–6	Disruption of ion balance across biological membranes by formation of complexes with lipid soluble cations	Cygro—5	1989
Monensin	Monovalent ionophore	100–110	Combines with sodium and potassium cations to form lipid-soluble complexes causing NA^+^ influx and disruption of membrane permeability to result in osmotic cell lysis	Elancoban—1	1971
				Coxidin—1	
				Monimax (with nicarbazin)—0	
Narasin	Monovalent ionophore	60–80	Forms dynamically reversible lipid soluble complexes with cations which alters transmembrane ion gradients and electrical potentials. Commonly used in combination with nicarbazin	Monteban—0	1986
				Maxiban (with nicarbazin)—0	
Salinomycin	Monovalent ionophore	44–66	Complexly binding monovalent cations, particularly potassium ions, promoting efflux into the cell mitochondria and cytoplasm	Sacox—0	1983
Semduramicin	Monovalent glycosidic ionophore	25	Disruption of ion balance across biological membranes by formation of complexes with lipid soluble cations	Aviax—5	1995
Decoquinate	Synthetic	30	Inhibition of the parasite mitochondrial electron transport and therefore respiration	Deccox—3	1967
Diclazuril	Synthetic	1	Interference in nucleic acid and parasite wall synthesis producing thickened incomplete oocyst walls, disruption of mitochondrial transmembrane potential	Coxiril—0	1990
				Clinacox—5	
Halofuginone	Synthetic	3	Unknown	Stenorol—5	1975
Nicarbazin	Synthetic	125	Acts as a calcium ionophore by increasing lipoprotein lipase activity, interferes with cholesterol metabolism and the formation of vitelline membrane. Commonly used in combination with narasin	Maxiban (with narasin)—0	1955
				Monimax (with monensin)—0	
Robenidine	Synthetic	33	Respiratory chain phosphorylation, ATPase and oxidative phosphorylation inhibition and energy metabolism interference, prevention of merozoite development	Robenz—5	1972

^a^Withdrawal periods vary between production systems with the majority of products not licenced for use in egg laying birds

### Advantages of chemoprophylaxis

Advantages of chemoprophylactic control of coccidiosis include the ease of administration. The majority of anticoccidial drugs are incorporated into milled feed or dispensed in the drinking water, providing direct and quick administration with no requirement for extra labour costs. Where chemoprophylactics are successfully used there is no need for treated birds to compete for energy with the parasite, therefore energy can be focussed into production gains. Reduced *Eimeria* cycling also reduces the risk of enteric dysbiosis and specific secondary bacterial enteritis, including for example necrotic enteritis for which uncontrolled infection with *Eimeria* species, especially *E. maxima* is a known predisposing factor (Al-Sheikhly and Al-Saieg 1980; Williams et al. 2003; Williams 2005; Adhikari et al. 2020). Furthermore, ionophores have been shown to have antimicrobial activity against gram positive bacteria including *Clostridium perfringens*, the causative agent of necrotic enteritis (Liu et al. 1976; Al-Sheikhly and Al-Saieg 1980; Williams et al. 2003; Williams 2005; Chapman et al. 2010; Lanckriet et al. 2010; Peek and Landman 2011; Adhikari et al. 2020). Finally, all seven established *Eimeria* species can be targeted with most chemoprophylactics, and it is likely that the three newly described *Eimeria* species will be equally susceptible.

### Disadvantages of chemoprophylaxis

Disadvantages of chemoprophylactic control most significantly include the widespread occurrence of anticoccidial drug resistance. First identified in the 1950s, resistance is now recognised in *Eimeria* against all current anticoccidial drugs where it can be defined as reduced effectiveness in comparison to efficacy at introduction, discussed further below (Cuckler and Malanga 1955; Joyner 1970; Braunius 1982; Chapman 1997; Chapman et al. 2013). Withdrawal periods are also a disadvantage for many products. Withdrawal of chemoprophylaxis is required during the period of greatest weight gain immediately before slaughter, leaving chickens vulnerable to uncontrolled infection. Additionally, consumer concerns over chemical residues in produce and consumer pressure for “drug-free”, particularly antibiotic free, production is a further disadvantage (Williams 1998; Jenkins 2004; Peek and Landman 2011; Kadykalo et al. 2018). Regulatory classification differences, mentioned earlier, poses another complication. For example, in the US ionophores are classified as antibiotics and are regulated as such, rather than as feed additives. In other countries, such as Sweden, prophylactic administration is banned for use in production systems (Remnant 2007; Swaggerty et al. 2011; Blake and Tomley 2014).

Finally, it is important to note the potential for environmental residues of anticoccidial drugs to pose toxicity risks to non-target-organisms. Research into this topic is based on data obtained from accidental ingestion or incorrect dosage ingestion case studies (Dorne et al. 2013; Mooney et al. 2020). Pathologically, ionophore toxicity causes increased mitochondrial uptake, and cardiac and peripheral muscle cell necrosis and clinical signs in animals and humans include muscle weakness, acute rhabdomyolysis and mucoid insufficiencies (Caldeira et al. 2001; Kouyoumdjian et al. 2001; Dorne et al. 2013). Synthetic anticoccidial drug toxicity, where studied, is variable depending on the drug and dose with effects ranging from vomiting (observed with decoquinate) to liver enlargement (observed with robenidine) and maternal and developmental toxicity (observed with halofuginone and nicarbazin) (Dorne et al. 2013; EFSA 2008a, b, c; 2019a; b).

### Development of resistance to anticoccidial drugs

Widespread use of anticoccidial drugs has increased *Eimeria* exposure to the compounds, providing multiple opportunities for resistance development. The World Health Organization (WHO) defines parasite resistance as: ‘the ability of a parasite strain to survive and/or multiply despite the administration and absorption of a drug given in doses equal to or higher than those usually recommended but within the limits of tolerance of the subject’ (WHO 1965; Abbas et al. 2011a; Peek and Landman 2011). Inherited resistance to anticoccidial drugs is a genetic adaptation to survive selection pressure applied by the specific mode of action of the compound(s) in an anticoccidial product. Acquiring partial (toleration of low concentrations) or complete resistance is complex and is defined by the degree of loss of sensitivity (Abbas et al. 2011a). Based on the characterisation of field strains it is thought that resistance to most anticoccidial drugs requires mutations at multiple loci, as opposed to arising from single point mutations, except for resistance to quinolones (Chapman 1997). However, the precise genetic basis of resistance is not known for any current anticoccidial drug. Cross-resistance has also been reported for some anticoccidial drugs, for example between the ionophores salinomycin, monensin, narasin, lasalocid and maduramicin, and between the synthetic compounds diclazuril and toltrazuril (Chapman 1997; Stephan et al. 1997; Abbas et al. 2008) which have closely related modes of action (Abbas et al. 2011a). The administration of inappropriate, specifically low, dosages are a significant factor in the development of resistance as this provides selection for partially resistant strains that become more prevalent in the absence of competition from susceptible strains, reducing efficacy of control and contributing to the step-wise development of completely resistant which then rapidly become the dominant population (Cuckler and Malanga 1955; Chapman 1997; Swaggerty et al. 2011). Incorrect dosing can sometimes result from accidental exposure of chickens to environmental residues. For example, close proximity to poultry farming activity has been found to increase the likelihood of detection of environmental drug residues in groundwater, potentially originating from excretion or spreading manure (Mooney et al. 2020). Additionally, poor poultry house hygiene can play a significant role in resistance development, as control of high level parasitaemia is difficult and resistance can spread rapidly once it arises.

### Future control by chemoprophylaxis

It is a testament to the poultry industry that anticoccidial chemoprophylactics are still effective, at any degree, based on the time elapsed since introduction and the high prevalence of resistance. Part of that success could be attributed to rotational use of different compounds between flocks, including selection of compounds with different modes of action to improve the likelihood of effective control against pre-existing resistant strains in the environment (Braunius 1982; Chapman 1997, 2007; Chapman et al. 2010; Noack et al. 2019). However, resistance remains a problem, demanding a range of alternative and combined approaches to control such as: vaccines and good husbandry measures, in addition to rotational use of anticoccidial drugs (Vegad 2004).

Research into new compounds for the control of coccidiosis has slowed in recent decades, predominantly due to lack of broad spectrum activity and genotoxicity, legislative restrictions, speed of resistance development and consumer concerns over chemical residues in food, all of which reduce incentive to discover and develop new anticoccidial compounds (Jenkins 2004; Biftu et al. 2006; Liang et al. 2007; Scribner et al. 2007, 2008; Peek and Landman 2011; Chapman et al. 2013; Kadykalo et al. 2018; Noack et al. 2019). When investigating potential new candidate compounds, an important consideration is parasite target stage, for example to target early asexual stages reducing pathology or by targeting gametes, preventing production of viable oocysts to reduce transmission.

Current research includes investigation of the anticoccidial effects of aminomizuril and ethanamizuril, both metabolites of nitromezuril; a triazine compound in the same family as toltrazuril and diclazuril. Both compounds were found to be effective against *E. tenella, E. necatrix, E. acervulina* and *E. maxima* with suggested action affecting transcription and protein metabolism including significant downregulation of GPI-linked surface antigen (SAG), proteins on the surface membranes of invasive sporozoites and merozoites thought to be related to host cell adhesion (Lal et al. 2009; Li et al. 2019; Noack et al. 2019; Zhang et al. 2019; Wang et al. 2020). The apparent lack of observed toxicity to the host and cross resistance with toltrazuril and diclazuril, encourage further development of nitromezuril and ethanamizuril as potential novel anticoccidials (Fei et al. 2013; Li et al. 2019; Zhang et al. 2020a,b). Future development and discovery of anticoccidial drugs is likely to lie closely with advances in genome annotation and understanding of parasite biology. An improved genome annotation and understanding of protein pathways could therefore provide opportunities for the identification of drug targets to interfere with parasite metabolism, survival and reproduction as a form of chemoprophylactic control.

## Vaccination

Anticoccidial vaccination aims to induce protective immunity against coccidiosis, traditionally viewed as the prevention of parasite replication and absence of clinical signs in birds challenged with *Eimeria* (Rose 1963; Beattie 1997). It has been known for decades that exposure to *Eimeria* oocysts, most notably multiple doses termed a “trickle infection”, can induce a robust protective immune response (Joyner and Norton 1976). Moreover, it is recognised that in the field for most species of *Eimeria* full flock immunity occurs only after chickens have experienced two or more cycles of infection (Chapman 1999). These observations form the basis of currently available live anticoccidial vaccines.

### Live and live attenuated vaccines

The first generation of vaccines against coccidiosis comprised admixtures of wild-type isolates of *Eimeria* oocysts and induced homologous immune protection against those species included in the mixture. Many wild-type vaccines have been developed, mainly using locally derived strains of parasites without any modification that changes their natural virulence. Thus, these vaccines remain fully virulent and are considered to be non-attenuated. Coccivac® containing *E. tenella* oocysts was launched as the first commercial coccidiosis vaccine in the US in 1952 under the trade name ‘DM Cecal Coccidiosis Vaccine’ (Edgar, 1958), Fig. 1. Thereafter, first-generation vaccines were developed to incorporate additional *Eimeria* species and have been widely utilized, particularly in North America (Soutter et al. 2020). Coccivac® has gone through many reformulations over the past six decades with variants of the original product: CocciVac-B, CocciVac-D, and most recently CocciVac D2. In 1985, Dr. Eng-Hong Lee (Lee 1987) developed Immucox, consisting of sporulated oocysts of *E. acervulina, E. tenella* and *E. maxima* with or without *E. necatrix* and *E. brunetti*. This was first marketed in Canada, but updated formulations are now used in more than 40 countries (Akanbi and Taiwo 2020).

**Fig. 1:**
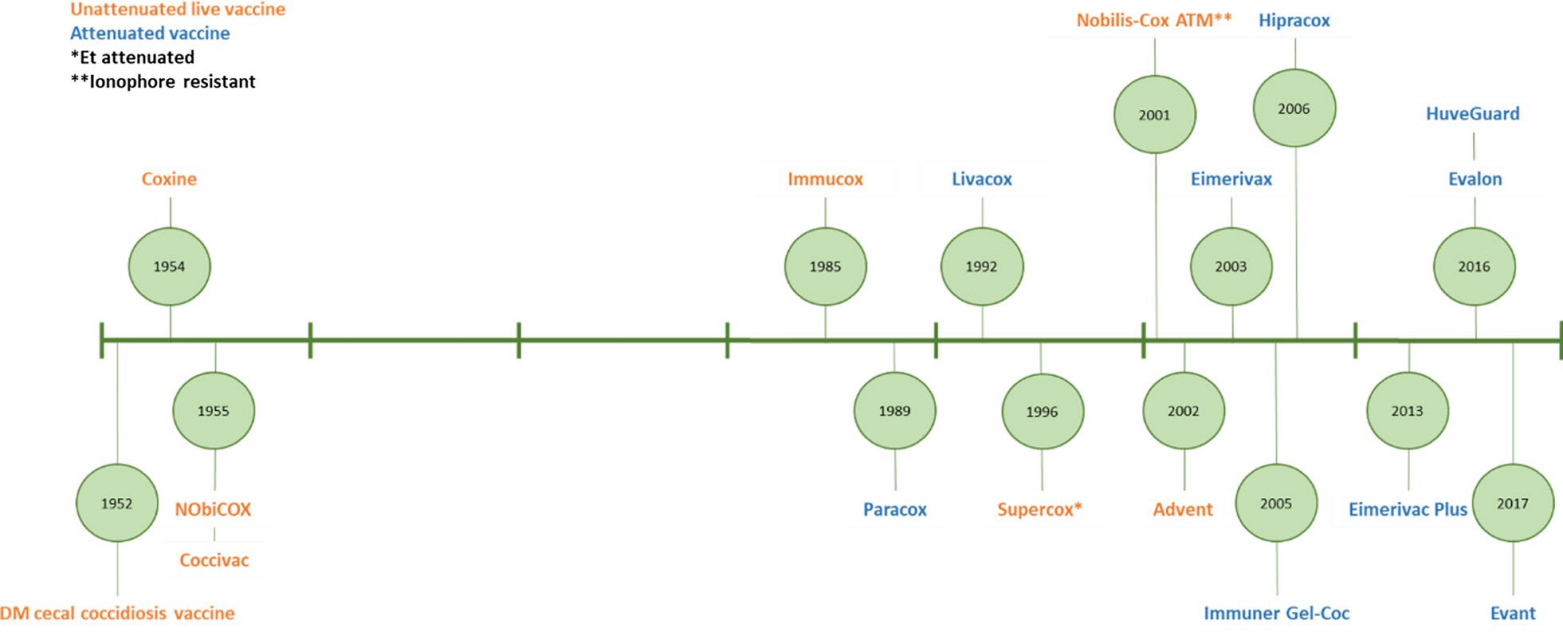
Timeline of vaccine development for use in chickens

Second generation vaccines contain live oocysts from attenuated lines of *Eimeria* parasites. Heat treatment and x-irradiation were used in attempts to attenuate *Eimeria*, but neither were fully successful to induce robust and reproducible preparations (Mielke 1993; Jungmann and Mielke 1989). In most cases, attenuation has been achieved by selection for a more rapidly completed life cycle. Repeatedly harvesting the earliest “precocious” oocysts produced at the beginning of the patent period can be used to select for stable *Eimeria* populations that have shorter life-cycles, fewer endogenous stages and reduced pathogenicity (Jeffers 1975). Importantly, these precocious parasites remain highly immunogenic. This approach has underpinned development of most commercial attenuated anticoccidial vaccines such as Paracox^®^, Eimerivax^®^, Hipracox^®^, Eimerivac Plus^®^ and Immuner Gel-Coc^®^. Although less common, attenuation has also been achieved by serial parasite passage through embryonated chicken eggs, for example selecting an *E. tenella* line that is included in the live attenuated Livacox^®^ vaccine range (McDougald and Jeffers 1976; Bedrnik et al. 1989; Shirley and Bedrník 1997).

Recent innovations in live anticoccidial vaccination include the development of vaccine series such as HuveGuard^®^, where single vaccination using the formulation MMAT (including *E. maxima*, *E. mitis*, *E. acervulina* and *E. tenella*) can induce protection against species that are especially relevant to broiler production. Subsequently, vaccination using HuveGuard^®^ NB from 14 days of age onwards can be used to vaccinate against *E. necatrix* and *E. brunetti*, less fecund species with longer lifecycles that are more relevant in older chickens. Alternatively, the vaccines Evalon^®^ and Evant^®^ produced by Hipra include a montanide-based adjuvant named Hipramune-T to enhance vaccine efficacy. In studies of *E. acervulina* and *E. tenella* profilin subunit antigen vaccines, montanide adjuvants have been shown to enhance protective immunity against avian coccidiosis by observed stimulation of IL-2, IL-10, IL-17A and IFN-γ gene transcription and increased CD8  +  lymphocyte infiltration at the site of immunization (Jang et al. 2010,2011a,b).

In contrast to the live non-attenuated vaccines, live attenuated anticoccidial vaccines have a far greater safety margin, even if administered at a ten-fold overdose. Nonetheless, both vaccine types are effective and can induce a significant degree of immune protection against *Eimeria* challenge. Currently, only live attenuated anticoccidial vaccines are licenced in Europe, in contrast to much of the rest of the World where live non-attenuated vaccines are more common. To date there is little evidence of parasite evolution towards resistance against vaccination, likely influenced by host exposure to the large and complex array of antigens expressed by *Eimeria* throughout their endogenous lifecycle (Shirley et al. 2005; Reid et al. 2014).

### Vaccination limitations

A significant drawback of live anticoccidial vaccines is that their production depends on in vivo growth of vaccinal parasites using chickens, as oocysts cannot be produced efficiently in vitro (Marugan-Hernandez et al. 2020). This is especially challenging for live-attenuated vaccine lines that have lowered reproductive capacity compared to non-attenuated equivalents, hence very large numbers of chickens are needed for vaccine production. Attenuated vaccines cost between two and six times more than non-attenuated alternatives (Blake et al. 2020). Immunity induced by live vaccination, as for natural infection, is exquisitely species-specific so effective vaccines have to include many different *Eimeria* species, and in some examples strains, each requiring independent amplification in chickens (Blake and Tomley 2014).

Differences between chicken production systems make it necessary to adapt vaccine formulations depending on the target animal. Those vaccines intended for use in intensively produced broiler chickens that are reared for only 5–7 weeks are likely to contain between three and five *Eimeria* species/strains, whereas vaccines for laying birds need to be more comprehensive and may contain all seven species of *Eimeria*. A critical inclusion for laying birds and breeding stock is *E. necatrix*, which can be a major cause of coccidiosis around the time when egg laying begins (McDougald et al. 1990).

Another complexity with these vaccines is the relative antigenic diversity that is observed in geographically distinct species or even strains of *Eimeria*. There is a risk of introducing an undesirable *Eimeria* species/strain present in vaccines into the farm environment. Strain-specific variation in *E. maxima*, the most antigenically diverse *Eimeria* species that infects chickens, has been reported. For example, it was found that the *E. maxima* parasites present in Immucox^®^ were unable to protect against an indigenous *E. maxima* strain isolated from a peninsula in the Eastern Shore of Maryland, USA (Danforth et al. 1997; Long and Millard 1979). In response, some formulations such as Paracox^®^ include two antigenically distinct *E. maxima* strains. Nonetheless, vaccines such as Paracox^®^ and Immucox^®^ are effective globally, indicating that antigenic diversity is not a common problem. However, no vaccine currently on the market includes any of the three *Eimeria* species described recently (Blake et al. 2021). These *Eimeria* species were first detected during an investigation of persistent vaccine failure (Morris et al. 2007), and all three have been shown to escape immunity induced by vaccination using at least one current vaccine (Blake et al. 2021). Careful evaluation is required when formulating live anticoccidial vaccines and those formulations may need to be fine-tuned according to experiences gained after implementation in new regions.

Application of non-attenuated live vaccines can pose safety issues if administered unevenly or to immune-suppressed chickens, resulting in compromised performance, clinical coccidiosis and even mortality (Anderson et al. 1976). Recent innovations in administration ameliorate this problem, as discussed below. Other limitations of live vaccines include the necessity for detailed quality control of the efficacy of each vaccine batch that can only be achieved in vivo, as well as a short shelf life and the requirement for a cold chain (Soutter et al. 2020). Another major challenge is inability to rapidly differentiate vaccinal *Eimeria* from field isolates, hindering quality control of vaccine administration, as well as diagnosis of vaccine-related problems and vaccine breaks.

### Vaccine administration

Several approaches are used to administer anticoccidial vaccines to chickens. Most common methods include spraying oocysts directly onto newly hatched chicks so that oocysts are ingested during preening (Chapman 1996; Albanese et al. 2018), spraying onto food, incorporating oocysts within peckable gels that are given to newly hatched chicks (Danforth et al. 1997; Danforth 1998) or by dispersal in drinking water with a viscous agent that keeps oocysts in suspension (Williams 1999). Oocyst vaccines can also be administered via eye-drop inoculation at the hatchery (Chapman 1996).

Alternatives include in ovo injection on the 18th day of egg incubation. In ovo vaccination is common for a range of viral vaccines and products such as Inovocox have been developed for in ovo vaccination against coccidiosis, currently used in the US. Such an approach is attractive for mass administration of an *Eimeria* vaccine (Watkins et al. 1995), offering the opportunity for efficient pre-hatch delivery prior to exposure to environmental challenge (Chapman et al. 2002). The requirement to introduce complex formulations of sterile oocysts has been challenging, but not unsustainable (Shirley et al. 2005).

Accurate and even vaccine application is important. Asynchronous exposure can result in significant variation in the number of oocysts ingested, causing uncontrolled variation in immune status and the possibility of a coccidiosis outbreak in chickens with low previous exposure and immune responses (Williams 1998). Poor litter management can exacerbate the problem, preventing ingestion of a sufficient number of vaccine oocysts and limiting oocyst cycling. Chickens may be subjected to relatively high challenge doses of non-attenuated oocysts, resulting in high pathogenicity, or even mortality, necessitating the use of therapeutic anticoccidial drugs following vaccination (Lightowlers 1994; Reid 1990). Mitigations of this problem have included the development of bioshuttle programmes where chickens are vaccinated with a non-attenuated drug-susceptible anticoccidial vaccine at or around day of hatch. Vaccinated chicks then receive a drug-free starter diet, permitting efficient vaccine replication, followed by routine chemoprophylactic supplementation of the grower diet to limit uncontrolled parasite reproduction. Thus, the efficacy and safety of live anticoccidial vaccines could be enhanced by careful adaptation of administration methods and housing techniques available to promote and then control oocyst cycling.

Benefits associated with use of live anticoccidial vaccines include reducing the selective pressure on parasites that favours anticoccidial drug resistance. Incorporating between three and five rounds of vaccination using drug-susceptible vaccine strains in an integrated coccidiosis control programme, interrupts the routine application of anticoccidial drugs, and can restore sensitivity to drugs such as salinomycin (Chapman and Jeffers 2015). Thus, rotating between anticoccidial control using live parasite vaccines and chemoprophylaxis can improve the longevity of current control measures.

### Future control by vaccination

Current limitations associated with chemoprophylaxis and vaccination for *Eimeria* have encouraged efforts to develop new control strategies, including a range of candidate recombinant vaccines. Since the first attempts to develop recombinant vaccines in the 1980’s several candidate antigens have been tried and tested (Vermeulen 1998; Blake and Tomley 2014), although no recombinant vaccine has reached the market. Nonetheless, efforts to develop recombinant vaccines are thought to be feasible because of the evident robust protective immune response achieved following natural *Eimeria* infection. Identification of antigens that induce natural immune protection can provide a rational basis to vaccine development. Several reviews focused on the identification, testing and delivery of immunoprotective antigens have been published in recent years (Blake et al. 2017; Venkatas and Adeleke 2019), so only brief details will be included here.

At least twenty five antigens have been defined and tested as vaccine candidates in *Eimeria* species, with varying levels of success (Blake et al. 2017). The results obtained from these studies typify the challenges faced by scientists trying to identify a “golden bullet” antigen or antigen cocktail to induce complete protection against complex pathogens such as *Eimeria*. One major issue is posed by the complexity of the *Eimeria* large genomes, with  ~ 6000–10,000 protein coding genes, dependant on species (ToxoDB 2021), making it difficult to predict genuinely protective antigens that stimulate an efficacious immune response. Vaccine development for other apicomplexan parasites such as *P. falciparum* and *T. gondii* have suffered similar frustrations (Takala and Plowe 2009; Arnott et al. 2014; Gedik et al. 2016). One explanation for this could be that, unlike live vaccines, recombinant vaccines expressing one or a small number of antigens induce a more focused and less reproducible or efficacious immune response. Recognising that natural anticoccidial immune responses are species-specific, and in some examples strain-specific, it is likely that multiple antigens will be required in a future recombinant vaccine. If one to three antigens are required to protect against a single *Eimeria* species (Blake et al. 2017), it is likely that a recombinant anticoccidial vaccine for broilers may require six or more antigens to protect against key species such as *E. acervulina*, *E. maxima* and *E. tenella*, incurring significant challenges for manufacture.

Another significant task remains identification of an efficient delivery system for optimised panels of vaccinal antigens. Observations that cellular immunity may be key to successful immune protection against *Eimeria* challenge suggest that vaccination should elicit a T-lymphocyte response (Lillehoj et al. 2005). DNA vaccination could be considered based on descriptions of the mode of immune stimulation (Kalinna 1997), although scalable delivery to large numbers of chickens remains problematic. Alternatives include vectored vaccine approaches, with examples including *Salmonella* strains (Konjufca et al. 2006) or various yeasts such as *Saccharomyces cerevisiae*, which can survive the gastrointestinal tract of the host and give rise to a mucosal immune response (Sun et al. 2014). Genetically modified *Eimeria* strains have been tested as vaccine vectors, inspired by the opportunity to deliver antigens to the target gut compartment in a relevant biological context. Although some studies have suggested promising results and feasibility, such as using transgenic *E. tenella* to deliver an immunogenic antigen of *E. maxima* to produce partial protective immunity against *E. maxima* challenge (Tang et al. 2018), there is, however, still some way to go before *Eimeria* can be used as an efficient vaccine vector (Pastor-Fernández et al. 2018). Ultimately, progress towards novel vaccines is likely to depend on a combination of a deeper understanding of the mechanisms of immunity induced by *Eimeria*, helping to select appropriate parasite antigens that can cover a wider range of strains, and an improvement of genetic and culture manipulation tools.

## Alternative strategies for control of coccidiosis

### Probiotic supplementation

Challenges to effective control of coccidiosis posed by resistance to chemoprophylaxis or limited availability to cost-effective vaccines have prompted exploration for alternative strategies (Gaggìa et al. 2010; Giannenas et al. 2012; Ritzi et al. 2014), identifying a range of probiotics and dietary supplements such as essential oils or other herbal products (Guo et al. 2004; Quiroz-Castañeda and Dantán-González 2015).

Probiotic additives are live non-pathogenic microorganisms that are considered to have a health benefit when administered to chickens, commonly via their diet, usually with the aim of improving and maintaining a healthy gut microbiome (Gaggìa et al. 2010; Giannenas et al. 2012; Ritzi et al. 2014). The school of thought behind the use of probiotic supplementation in the control of coccidiosis is that a healthy microbiota can play a role in host immune system enhancement and protection against some intestinal pathogens (Dalloul et al. 2005; Ritzi et al. 2014). The most commonly used probiotics in the livestock industry, including in poultry are: *Bacillus, Bifidobacteria, Enterococcus*, *Lactobacillus* and *Saccharomyces* (Gaggìa et al. 2010). The efficacy of probiotics in protection against pathological lesions caused by *Eimeria* species has not been definitively proven (Giannenas et al. 2012). Some studies have shown that treatment with probiotics, such as *Lactobacillus salivarius and L. acidophilus*, associates with reduced oocyst shedding, while supplementation with a *Bacillus* has been associated with lower lesion scores compared to untreated controls (Dalloul et al. 2003, 2005; Tierney et al. 2004; El-Dakhly et al. 2006; Lee et al. 2010b; Giannenas et al. 2012). Typically, studies into the efficacy of probiotics conclude that they can alleviate the effects of coccidiosis when anticoccidial drugs are not in use however when performance is directly compared, anticoccidial drugs tend to outperform probiotic treatment, particularly at peak infection and in measures such as oocyst shedding or feed conversion ratio (Giannenas et al. 2012; Bozkurt et al. 2014; Ritzi et al. 2014).

### Essential oil and organic acid supplementation

Several essential oils have also been suggested as alternatives for the control of coccidiosis, commonly due to their reported antiparasitic action. Some, such as oregano, thyme and garlic, have been associated with reduced disease burden in terms of improved body weight gain, reduced oocyst shedding following challenge and fewer pathological lesions (Giannenas et al. 2003; Küçükyilmaz et al. 2012; Abou-Elkhair et al. 2014). The efficacy of essential oils is not, however, well characterised. Generally, the use of anticoccidial drugs outperforms treatment with essential oils and in some cases toxicity of essential oils used can result in poor performance, indicating the importance of establishing an effective concentration (Giannenas et al. 2003; Christaki et al. 2004; Oviedo-Rondón et al. 2005, 2006; Küçükyilmaz et al. 2012). Essential oils may therefore be a useful supplement for chicken diets to provide some anticoccidial effects and improve host intestinal health, although they are unlikely to replace anticoccidial drugs.

Dietary supplementation with organic acids such as acetic and butyrate acid have also been suggested for the control of coccidiosis due to observed improved weight gain, feed conversion ratio and reduced oocyst shedding and lesion scores, in addition to their growth-promoting, antimicrobial and immune stimulating properties (Abbas et al. 2011b; Ali et al. 2014). In some studies reduced feed intake has been reported due to reduced palatability of feed supplemented with organic acids (Cave 1984; Ali et al. 2014), therefore when supplementing feeds it is important to consider the effect on the feed acidity and odour and minimise adverse changes that would reduce feed intake or body weight gain.

### Future control by alternative measures

For many of the alternative measures suggested for the control of coccidiosis there is a lack of understanding of the full mechanism of action against the parasite. Additionally, in most cases the greatest positive effects against infection with *Eimeria* were observed when the alternative measure was used in combination with anticoccidial drugs or alongside vaccination (Abbas et al. 2011b; Giannenas et al. 2012; Ali et al. 2014; Bozkurt et al. 2014; Ritzi et al. 2014). It is therefore important to note that further investigation is required into these alternative measures before conclusions can be drawn about their cost effectiveness in comparison to other current measures for control.

## Conclusions

Successful control of coccidiosis is multifactorial. Good animal husbandry is a key cornerstone in this endeavour and involves strict biosecurity measures, commonly supplemented with chemoprophylaxis and/or vaccination (Awais et al. 2012; Reid et al. 2014; Lawal et al. 2016; Morgan and Godwin 2017). The outcome of control by chemoprophylaxis or vaccination is influenced by factors such as chicken age, type of production system and genetic capacity for tolerance to subclinical infection.

Alternative control measures including probiotics and a range of food supplements are becoming increasingly popular, however, evidence of their efficacy remains limited. Improved control can be supported through better education and management of potential risk factors, for example: temperature, humidity, accumulation of sporulated oocysts on litter and rotational use of chemoprophylactics to reduce resistance emergence.

Immunity induced by current anticoccidial vaccines is *Eimeria* species-specific, and vaccine composition must be tailored to each geographical region and chicken production system (Graat et al. 1994), therefore, accurately identifying geographical prevalence and genetic diversity within *Eimeria* species and strains is important for successful control (Morris and Gasser 2006; Lee et al. 2010a; Ogedengbe et al. 2011; Györke et al. 2013).

In the future, improved and more readily scalable vaccines can be expected to make a bigger contribution to control of coccidiosis, together with complementary strategies such as rotational use of anticoccidial drugs with differing modes of action and selective breeding for improved resistance to the parasite. As the research community continues to increase understanding of *Eimeria* species parasites and host immunity, control measures will develop through identification of anticoccidial drug and vaccine candidate targets. These efforts can improve chicken welfare and reduce economic losses incurred by the poultry industry as a whole, including in low- and middle-income countries vulnerable to the economic burden of coccidiosis.

## Data Availability

Not applicable.

## References

[CR1] Abbas R, Iqbal Z, Sindhu Z-D, Khan M, Arshad M (2008). Identification of cross-resistance and multiple resistance in *Eimeria*
*tenella* field isolates to commonly used anticoccidials in Pakistan. J Appl Poult Res.

[CR2] Abbas R, Iqbal Z, Blake D, Khan M, Saleemi M (2011). Anticoccidial drug resistance in fowl coccidia: the state of play revisited. Worlds Poult Sci J.

[CR3] Abbas RZ, Munawar SH, Manzoor Z, Iqbal Z, Khan MN, Saleemi MK, Zia MA, Yousaf A (2011). Anticoccidial effects of acetic acid on performance and pathogenic parameters in broiler chickens challenged with *Eimeria*
*tenella*. Pesqui Vet Brasil.

[CR4] Abou-Elkhair R, Gaafar KM, Elbahy N, Helal MA, Mahboub HD, Sameh G (2014). Bioactive effect of dietary supplementation with essential oils blend of oregano, thyme and garlic oils on performance of broilers infected with *Eimeria* species. Glob Vet.

[CR5] Adhikari P, Kiess A, Adhikari R, Jha R (2020). An approach to alternative strategies to control avian coccidiosis and necrotic enteritis. J Appl Poult Res.

[CR6] Ahad S, Tanveer S, Malik TA (2015). Seasonal impact on the prevalence of coccidian infection in broiler chicks across poultry farms in the Kashmir valley. J Parasit Dis.

[CR7] Akanbi OB, Taiwo VO (2020). The effect of a local isolate and Houghton strain of *Eimeria*
*tenella* on clinical and growth parameters following challenge in chickens vaccinated with IMMUCOX^®^ and LIVACOX^®^ vaccines. J Parasit Dis.

[CR8] Albanese GA, Tensa LR, Aston EJ, Hilt DA, Jordan BJ (2018). Evaluation of a coccidia vaccine using spray and gel applications. Poult Sci.

[CR9] Al-Gawad AA, Mahdy OA, El-Massry AA, Al-Aziz MS (2012). Studies on coccidia of Egyptian Balady breed chickens. Life Sci J.

[CR10] Ali A, Seddiek SA, Khater H (2014). Effect of butyrate, clopidol and their combination on the performance of broilers infected with *Eimeria*
*maxima*. Br Poult Sci.

[CR11] Allen PC, Fetterer R (2002). Recent advances in biology and immunobiology of *Eimeria* species and in diagnosis and control of infection with these coccidian parasites of poultry. Clin Microbiol Rev.

[CR12] Al-Sheikhly F, Al-Saieg A (1980). Role of coccidia in the occurrence of necrotic enteritis of chickens. Avian Dis.

[CR13] Anadón A, Martínez-Larrañaga M. Veterinary drugs residues: coccidiostats. In: Motarjemi Y, editor. Encyclopedia of food safety; 2014;3:63-75.

[CR14] Anderson WI, Reid WM, Johnson JK (1976). Effects of high environmental temperatures on cecal coccidiosis. Poult Sci.

[CR15] AnimalDrugs@FDA. US food and drug. 2021. https://animaldrugsatfda.fda.gov/adafda/views/#/home/searchResult.

[CR16] Arnott A, Wapling J, Mueller I, Ramsland PA, Siba PM, Reeder JC, Barry AE (2014). Distinct patterns of diversity, population structure and evolution in the AMA1 genes of sympatric *Plasmodium*
*falciparum* and *Plasmodium*
*vivax* populations of Papua New Guinea from an area of similarly high transmission. Malar J.

[CR17] Awais MM, Akhtar M, Iqbal Z, Muhammad F, Anwar MI (2012). Seasonal prevalence of coccidiosis in industrial broiler chickens in Faisalabad, Punjab, Pakistan. Trop Anim Health Prod.

[CR18] Bacciu N, Bed’Hom B, Filangi O, Romé H, Gourichon D, Répérant J-M, Le Roy P, Pinard-van der Laan M-H, Demeure O (2014). QTL detection for coccidiosis (*Eimeria*
*tenella*) resistance in a Fayoumi×Leghorn F2 cross, using a medium-density SNP panel. Genet Sel Evol.

[CR19] Beattie SE (1997). Immunity to and transport of sporozoites of *Eimeria* species in the domestic fowl, *Gallus**domesticus*.

[CR20] Bedrnik P, Kucera J, Firmanova A, Jurkovic P (1989). Field vaccination of broilers against coccidiosis. Avian Pathol.

[CR21] Belanger A, Haydon K, Keffaber K, Marsteller T (2013). The science and mechanisms behind ionophores for pigs and poultry.

[CR22] Berger J, Rachlin A, Scott W, Sternbach L, Goldberg M (1951). The isolation of three new crystalline antibiotics from streptomyces1. J Am Chem Soc.

[CR23] Biftu T, Feng D, Fisher M, Liang G-B, Qian X, Scribner A, Dennis R, Lee S, Liberator PA, Brown C (2006). Synthesis and SAR studies of very potent imidazopyridine antiprotozoal agents. Bioorg Med Chem Lett.

[CR24] Bishop SC, Woolliams JA (2014). Genomics and disease resistance studies in livestock. Livest Sci.

[CR25] Blake DP, Tomley FM (2014). Securing poultry production from the ever-present *Eimeria* challenge. Trends Parasitol.

[CR26] Blake DP, Hesketh P, Archer A, Carroll F, Shirley MW, Smith AL (2005). The influence of immunizing dose size and schedule on immunity to subsequent challenge with antigenically distinct strains of *Eimeria*
*maxima*. Avian Pathol.

[CR27] Blake DP, Clark EL, Macdonald SE, Thenmozhi V, Kundu K, Garg R, Jatau ID, Ayoade S, Kawahara F, Moftah A (2015). Population, genetic, and antigenic diversity of the apicomplexan *Eimeria*
*tenella* and their relevance to vaccine development. Proc Natl Acad Sci.

[CR28] Blake DP, Pastor-Fernández I, Nolan MJ, Tomley FM (2017). Recombinant anticoccidial vaccines-a cup half full?. Infect Genet Evol.

[CR29] Blake DP, Knox J, Dehaeck B, Huntington B, Rathinam T, Ravipati V, Ayoade S, Gilbert W, Adebambo AO, Jatau ID (2020). Re-calculating the cost of coccidiosis in chickens. Vet Res.

[CR30] Blake D, Vrba V, Xia D, Danladi Jatau I, Spiro S, Nolan MJ, Underwood G, Tomley F (2021). Genetic and biological characterisation of three cryptic Eimeria operational taxonomic units that infect chickens (*Gallus*
*gallus*
*domesticus*). Int J Parasitol.

[CR31] Boulton K, Nolan MJ, Wu Z, Psifidi A, Riggio V, Harman K, Bishop SC, Kaiser P, Abrahamsen MS, Hawken R (2018). Phenotypic and genetic variation in the response of chickens to *Eimeria*
*tenella* induced coccidiosis. Genet Sel Evol.

[CR32] Boulton K, Nolan MJ, Wu Z, Riggio V, Matika O, Harman K, Hocking PM, Bumstead N, Hesketh P, Archer A (2018). Dissecting the genomic architecture of resistance to *Eimeria*
*maxima* parasitism in the chicken. Front Genet.

[CR33] Bozkurt M, Giannenas I, Küçükyilmaz K, Christaki E, Florou-Paneri P (2013). An update on approaches to controlling coccidia in poultry using botanical extracts. Br Poult Sci.

[CR34] Bozkurt M, Aysul N, Küçükyilmaz K, Aypak S, Ege G, Catli A, Akşit H, Çöven F, Seyrek K, Çınar M (2014). Efficacy of in-feed preparations of an anticoccidial, multienzyme, prebiotic, probiotic, and herbal essential oil mixture in healthy and *Eimeria* spp.-infected broilers. Poult sci.

[CR35] Braunius W (1982). Coccidiosis in broilers: the effective use of anticoccidial drugs. Worlds Poult Sci J.

[CR36] Bumstead N, Millard B (1992). Variation in susceptibility of inbred lines of chickens to seven species of *Eimeria*. Parasitology.

[CR37] Burrell A, Tomley FM, Vaughan S, Marugan-Hernandez V (2020). Life cycle stages, specific organelles and invasion mechanisms of *Eimeria* species. Parasitology.

[CR38] Caldeira C, Neves WS, Cury PM, Serrano P, Baptista MA, Burdmann EA (2001). Rhabdomyolysis, acute renal failure, and death after monensin ingestion. Am J Kidney Dis.

[CR39] Cantacessi C, Riddell S, Morris GM, Doran T, Woods WG, Otranto D, Gasser RB (2008). Genetic characterization of three unique operational taxonomic units of *Eimeria* from chickens in Australia based on nuclear spacer ribosomal DNA. Vet Parasitol.

[CR40] Castanon J (2007). History of the use of antibiotic as growth promoters in European poultry feeds. Poult Sci.

[CR41] Cave N (1984). Effect of dietary propionic and lactic acids on feed intake by chicks. Poult Sci.

[CR42] Champion LR (1954). The inheritance of resistance to cecal coccidiosis in the domestic fowl. Poult Sci.

[CR43] Chapman HD (1996). Administration of a coccidiosis vaccine to day-old turkeys via the eye and development of immunity to *Eimeria* species. Poult Sci.

[CR44] Chapman H (1997). Biochemical, genetic and applied aspects of drug resistance in *Eimeria* parasites of the fowl. Avian Pathol.

[CR45] Chapman H (1999). The development of immunity to *Eimeria* species in broilers given anticoccidial drugs. Avian Pathol.

[CR46] Chapman H (2001). Use of anticoccidial drugs in broiler chickens in the USA: analysis for the years 1995 to 1999. Poult Sci.

[CR47] Chapman HD (2005). Perspectives for the control of coccidiosis in poultry by chemotherapy and vaccination.

[CR48] Chapman H (2007). Rotation programmes for coccidiosis control. Int Poult Product.

[CR49] Chapman H (2009). A landmark contribution to poultry science—prophylactic control of coccidiosis in poultry. Poult Sci.

[CR50] Chapman H, Jeffers T (2015). Restoration of sensitivity to salinomycin in *Eimeria* following 5 flocks of broiler chickens reared in floor-pens using drug programs and vaccination to control coccidiosis. Poult Sci.

[CR51] Chapman H, Cherry T, Danforth H, Richards G, Shirley M, Williams R (2002). Sustainable coccidiosis control in poultry production: the role of live vaccines. Int J Parasitol.

[CR52] Chapman H, Jeffers T, Williams R (2010). Forty years of monensin for the control of coccidiosis in poultry. Poult Sci.

[CR53] Chapman HD, Barta JR, Blake D, Gruber A, Jenkins M, Smith NC, Suo X, Tomley FM (2013). A selective review of advances in coccidiosis research. Adv Parasitol.

[CR54] Christaki E, Florou-Paneri P, Giannenas I, Papazahariadou M, Botsoglou NA, Spais AB (2004). Effect of a mixture of herbal extracts on broiler chickens infected with *Eimeria*
*tenella*. Anim Res.

[CR55] Cisman M, Ahmed Z, Mohamoud H (2020). Scope specification of coccidiosis in the poultry on researchers. Int J Avian Wildl Biol.

[CR56] Clark EL, Macdonald SE, Thenmozhi V, Kundu K, Garg R, Kumar S, Ayoade S, Fornace KM, Jatau ID, Moftah A (2016). Cryptic *Eimeria* genotypes are common across the Southern but not Northern hemisphere. Int J Parasitol.

[CR57] Clarke L, Fodey TL, Crooks SR, Moloney M, O'Mahony J, Delahaut P, O’Kennedy R, Danaher M (2014). A review of coccidiostats and the analysis of their residues in meat and other food. Meat Sci.

[CR58] Cuckler AC, Malanga CM (1955). Studies on drug resistance in coccidia. J Parasitol.

[CR59] Dalloul R, Lillehoj H, Shellem T, Doerr J (2003). Enhanced mucosal immunity against *Eimeria*
*acervulina* in broilers fed a *Lactobacillus*-based probiotic. Poult Sci.

[CR60] Dalloul RA, Lillehoj HS, Tamim NM, Shellem TA, Doerr JA (2005). Induction of local protective immunity to *Eimeria*
*acervulina* by a Lactobacillus-based probiotic. Comp Immunol Microbiol Infect Dis.

[CR61] Danforth HD (1998). Use of live oocyst vaccines in the control of avian coccidiosis: experimental studies and field trials. Int J Parasitol.

[CR62] Danforth HD, Lee EH, Martin A, Dekich M (1997). Evaluation of a gel-immunization technique used with two different Immucox vaccine formulations in battery and floor-pen trials with broiler chickens. Parasitol Res.

[CR63] Dar AS, Anwar AH (1981). Incidence and pathogenesis of coccidiosis in chickens around Faisalabad. Pak Vet J.

[CR64] Dorne J, Fernández-Cruz M, Bertelsen U, Renshaw D, Peltonen K, Anadon A, Feil A, Sanders P, Wester P, Fink-Gremmels J (2013). Risk assessment of coccidostatics during feed cross-contamination: animal and human health aspects. Toxicol Appl Pharmacol.

[CR65] Dubey JP (2019). Coccidiosis in livestock, poultry, companion animals, and humans.

[CR66] Edgar S (1955). Sporulation of oocysts at specific temperatures and notes on the prepatent period of several species of avian coccidia. J Parasitol.

[CR67] EFSA, E. F. S. A. (2008). Cross-contamination of non-target feedingstuffs by decoquinate authorised for use as a feed additive-scientific opinion of the panel on contaminants in the food chain. EFSA J.

[CR68] EFSA, E. F. S. A. (2008). Cross-contamination of non-target feedingstuffs by halofuginone hydrobromide authorised for use as a feed additive-scientific opinion of the panel on contaminants in the food chain. EFSA J.

[CR69] EFSA, E. F. S. A. (2008). Cross-contamination of non-target feedingstuffs by nicarbazin authorised for use as a feed additive-scientific opinion of the panel on contaminants in the food chain. EFSA J.

[CR70] EFSA, E. F. S. A. (2019). Safety and efficacy of Deccox^®^(decoquinate) for chickens for fattening. EFSA J.

[CR71] EFSA, E. F. S. A. (2019). Safety and efficacy of Robenz^®^ 66G (robenidine hydrochloride) for chickens for fattening and turkeys for fattening. EFSA J.

[CR72] El-Dakhly KM, El-Sawah AA, Shalaby A, El-Nesr KA (2006). The efficacy of *Lactobacillus*
*acidophilus* and/or diclazuril for inhibition and control of *Eimeria*
*tenella* infection in balady chicks. Kafrelsheikh Vet Med J.

[CR73] Etuk E, Okoli I, Uko M (2004). Prevalence and management issues associated with poultry coccidiosis in Abak agricultural zone of Akwa Ibom state, Nigeria. Int J Poult Sci.

[CR74] EU (2021). European Union Register of Feed Additives pursuant to Regulation (EC) No. 1831/2003, Annex I: list of additives.

[CR75] Farr MM, Wehr EE (1949). Survival of *Eimeria*
*acervulina*, *E.*
*tenella*, and *E.*
*maxima* oocysts on soil under various field conditions. Ann NY Acad Sci.

[CR76] Fatoba AJ, Adeleke MA (2018). Diagnosis and control of chicken coccidiosis: a recent update. J Parasit Dis.

[CR77] Fayer R (1980). Epidemiology of protozoan infections: the coccidia. Vet Parasitol.

[CR78] Fei C, Fan C, Zhao Q, Lin Y, Wang X, Zheng W, Wang M, Zhang K, Zhang L, Li T (2013). Anticoccidial effects of a novel triazine nitromezuril in broiler chickens. Vet Parasitol.

[CR79] Fish F (1931). The effect of physical and chemical agents on the oocysts of *Eimeria*
*tenella*. Science.

[CR80] Fornace KM, Clark EL, Macdonald SE, Namangala B, Karimuribo E, Awuni JA, Thieme O, Blake DP, Rushton J (2013). Occurrence of *Eimeria* species parasites on small-scale commercial chicken farms in Africa and indication of economic profitability. PLoS ONE.

[CR81] Gaggìa F, Mattarelli P, Biavati B (2010). Probiotics and prebiotics in animal feeding for safe food production. Int J Food Microbiol.

[CR82] Gedik Y, İz SG, Can H, Döşkaya AD, Gürhan SİD, Gürüz Y, Döşkaya M (2016). Immunogenic multistage recombinant protein vaccine confers partial protection against experimental toxoplasmosis mimicking natural infection in murine model. Trials Vaccinol.

[CR83] Gerhold RWJ. Overview of coccidiosis in poultry. 2014. https://www.msdvetmanual.com/poultry/coccidiosis/overview-of-coccidiosis-in-poultry. Accessed 12 July 2021.

[CR84] Giannenas I, Florou-Paneri P, Papazahariadou M, Christaki E, Botsoglou N, Spais A (2003). Effect of dietary supplementation with oregano essential oil on performance of broilers after experimental infection with *Eimeria*
*tenella*. Arch Anim Nutr.

[CR85] Giannenas I, Papadopoulos E, Tsalie E, Triantafillou E, Henikl S, Teichmann K, Tontis D (2012). Assessment of dietary supplementation with probiotics on performance, intestinal morphology and microflora of chickens infected with *Eimeria*
*tenella*. Vet Parasitol.

[CR86] Goetting V, Lee K, Tell LA (2011). Pharmacokinetics of veterinary drugs in laying hens and residues in eggs: a review of the literature. J Vet Pharmacol Ther.

[CR87] Graat E, Henken A, Ploeger H, Noordhuizen J, Vertommen M (1994). Rate and course of sporulation of oocysts of *Eimeria*
*acervulina* under different environmental conditions. Parasitology.

[CR88] Gross W (1985). Effect of social environment and oocyst dose on resistance and immunity to *Eimeria*
*tenella* challenge. Avian Dis.

[CR89] Grumbles L, Delaplane J, Higgins T (1948). Continuous feeding of low concentrations of sulfaquinoxaline for the control of coccidiosis in poultry. Poult Sci.

[CR90] Guo F, Kwakkel R, Williams B, Parmentier H, Li W, Yang Z, Verstegen M (2004). Effects of mushroom and herb polysaccharides on cellular and humoral immune responses of *Eimeria*
*tenella*-infected chickens. Poult Sci.

[CR91] Györke A, Pop L, Cozma V (2013). Prevalence and distribution of *Eimeria* species in broiler chicken farms of different capacities. Parasite.

[CR92] Hafez HM (2008). Poultry coccidiosis: prevention and control approaches. Archiv Fur Geflugelkd.

[CR93] Hamzić E, Buitenhuis B, Hérault F, Hawken R, Abrahamsen MS, Servin B, Elsen J-M, Pinard-van der Laan M-H, Bed’Hom B (2015). Genome-wide association study and biological pathway analysis of the *Eimeria* maxima response in broilers. Genet Sel Evol.

[CR94] Harder A, Haberkorn A (1989). Possible mode of action of toltrazuril: studies on two *Eimeria* species and mammalian and *Ascaris*
*suum* enzymes. Parasitol Res.

[CR95] Hauck R, Carrisosa M, McCrea BA, Dormitorio T, Macklin KS (2019). Evaluation of next-generation amplicon sequencing to identify *Eimeria* spp. of chickens. Avian Dis.

[CR96] Haug A, Gjevre A-G, Thebo P, Mattsson JG, Kaldhusdal M (2008). Coccidial infections in commercial broilers: epidemiological aspects and comparison of *Eimeria* species identification by morphometric and polymerase chain reaction techniques. Avian Pathol.

[CR97] Hinsu AT, Thakkar JR, Koringa PG, Vrba V, Jakhesara SJ, Psifidi A, Guitian J, Tomley FM, Rank DN, Raman M (2018). Illumina next generation sequencing for the analysis of *Eimeria* populations in commercial broilers and indigenous chickens. Front Vet Sci.

[CR98] Horton-Smith C, Long P (1965). The development of *Eimeria*
*necatrix* Johnson, 1930 and *Eimeria*
*brunetti* Levine, 1942 in the caeca of the domestic fowl (*Gallus*
*domesticus*). Parasitology.

[CR99] Horton-Smith C, Taylor E, Turtle E (1940). Ammonia fumigation for coccidial disinfection. Vet Rec.

[CR100] Jang SI, Lillehoj HS, Lee SH, Lee KW, Park MS, Bauchan GR, Lillehoj EP, Bertrand F, Dupuis L, Deville S (2010). Immunoenhancing effects of Montanide™ ISA oil-based adjuvants on recombinant coccidia antigen vaccination against *Eimeria* acervulina *infection*. Vet Parasitol.

[CR101] Jang SI, Lillehoj HS, Lee SH, Lee KW, Lillehoj EP, Bertrand F, Dupuis L, Deville S (2011). Montanide™ ISA 71 VG adjuvant enhances antibody and cell-mediated immune responses to profilin subunit antigen vaccination and promotes protection against *Eimeria*
*acervulina* and *Eimeria*
*tenella*. Exp Parasitol.

[CR102] Jang SI, Lillehoj HS, Lee SH, Lee KW, Lillehoj EP, Bertrand F, Dupuis L, Deville S (2011). Mucosal immunity against *Eimeria*
*acervulina* infection in broiler chickens following oral immunization with profilin in Montanide™ adjuvants. Exp Parasitol.

[CR103] Jeffers T (1975). Attenuation of *Eimeria*
*tenella* through selection for precociousness. J parasitol.

[CR104] Jeffers T, Challey J, McGibbon W (1970). Response of several lines of fowl and their single-cross progeny to experimental infection with *Eimeria*
*tenella*. Avian Dis.

[CR105] Jenkins M (2004). Control of avian coccidiosis: drugs and vaccines.

[CR106] Joyner L (1958). Experimental *Eimeria*
*mitis* infections in chickens. Parasitology.

[CR107] Joyner L (1970). Coccidiosis: problems arising from the development of anticoccidial drug resistance. Exp Parasitol.

[CR108] Joyner L, Davies S (1960). Detection and assessment of sublethal infections of *Eimeria*
*tenella* and *Eimeria*
*necatrix*. Exp Parasitol.

[CR109] Joyner LP, Norton CC (1976). The immunity arising from continuous low-level infection with *Eimeria*
*maxima* and *Eimeria*
*acervulina*. Parasitology.

[CR110] Jungmann R, Mielke D (1989). Use of *Eimeria*
*tenella* radiovaccine for immunoprophylaxis in fowl against coccidiosis. Monatshefte Fuer Vet.

[CR111] Kadykalo S, Roberts T, Thompson M, Wilson J, Lang M, Espeisse O (2018). The value of anticoccidials for sustainable global poultry production. Int J Antimicrob Agents.

[CR112] Kalinna BH (1997). DNA vaccines for parasitic infections. Immunol Cell Biol.

[CR113] Kant V, Singh P, Verma PK, Bais I, Parmar MS, Gopal A, Gopal V (2013). Anticoccidial drugs used in the poultry: an overview. Sci Int.

[CR114] Khan M, Irshad H, Anjum R, Jahangir M, Nasir U (2006). Eimeriosis in poultry of Rawalpindi/Islamabad area. Pak Vet J.

[CR115] Kim D, Hong Y, Park D, Lamont S, Lillehoj H, Pinard MH, Gay C, Pastoret PP, Dodet B (2008). Differential immune-related gene expression in two genetically disparate chicken lines during infection by emopenEimeriaemclose emopenmaximaemclose. Animal genomics for animal health.

[CR116] Konjufca V, Wanda SY, Jenkins MC, Curtiss R (2006). A recombinant attenuated *Salmonella*
*enterica* serovar Typhimurium vaccine encoding *Eimeria*
*acervulina* antigen offers protection against *E.*
*acervulina* challenge. Infect Immun.

[CR117] Kouyoumdjian JA, Morita MDPA, Sato AK, Pissolatti AF (2001). Fatal rhabdomyolysis after acute sodium monensin (Rumensin^®^) toxicity: case report. Arq Neuropsiquiatr.

[CR118] Küçükyilmaz K, Bozkurt M, Selek N, Güven E, Eren H, Atasever A, Bintaş E, Çatlı AU, Çınar M (2012). Effects of vaccination against coccidiosis, with and without a specific herbal essential oil blend, on performance, oocyst excretion and serum IBD titers of broilers reared on litter. Ital J Anim Sci.

[CR119] Lai L, Bumstead J, Liu Y, Garnett J, Campanero-Rhodes MA, Blake DP, Palma AS, Chai W, Ferguson DJ, Simpson P (2011). The role of sialyl glycan recognition in host tissue tropism of the avian parasite *Eimeria*
*tenella*. PLoS Pathog.

[CR120] Lal K, Bromley E, Oakes R, Prieto JH, Sanderson SJ, Kurian D, Hunt L, Yates JR, Wastling JM, Sinden RE (2009). Proteomic comparison of four *Eimeria*
*tenella* life-cycle stages: unsporulated oocyst, sporulated oocyst, sporozoite and second-generation merozoite. Proteomics.

[CR121] Lanckriet A, Timbermont L, De Gussem M, Marien M, Vancraeynest D, Haesebrouck F, Ducatelle R, Van Immerseel F (2010). The effect of commonly used anticoccidials and antibiotics in a subclinical necrotic enteritis model. Avian Pathol.

[CR122] Lawal JR, Jajere SM, Ibrahim UI, Geidam YA, Gulani IA, Musa G, Ibekwe BU (2016). Prevalence of coccidiosis among village and exotic breed of chickens in Maiduguri, Nigeria. Vet World.

[CR123] Lee EH (1987). Vaccination against coccidiosis in commercial roaster chickens. Can Vet J.

[CR124] Lee BH, Kim WH, Jeong J, Yoo J, Kwon Y-K, Jung BY, Kwon JH, Lillehoj HS, Min W (2010). Prevalence and cross-immunity of *Eimeria* species on Korean chicken farms. J Vet Med Sci.

[CR125] Lee K-W, Lillehoj HS, Jang SI, Li G, Lee S-H, Lillehoj EP, Siragusa GR (2010). Effect of *Bacillus*-based direct-fed microbials on *Eimeria* maxima infection in broiler chickens. Comp Immunol Microbiol Infect Dis.

[CR126] Li X-Y, Liu L-L, Zhang M, Zhang L-F, Wang X-Y, Wang M, Zhang K-Y, Liu Y-C, Wang C-M, Xue F-Q (2019). Proteomic analysis of the second-generation merozoites of *Eimeria*
*tenella* under nitromezuril and ethanamizuril stress. Parasites Vectors.

[CR127] Liang G-B, Qian X, Feng D, Fisher M, Brown CM, Gurnett A, Leavitt PS, Liberator PA, Misura AS, Tamas T (2007). Synthesis and SAR studies of potent imidazopyridine anticoccidial agents. Bioorg Med Chem Lett.

[CR128] Lightowlers M (1994). Vaccination against animal parasites. Vet Parasitol.

[CR129] Lillehoj HS, Ding X, Quiroz MA, Bevensee E, Lillehoj EP (2005). Resistance to intestinal coccidiosis following DNA immunization with the cloned 3–1E *Eimeria* gene plus IL-2, IL-15, and IFN-γ. Avian Dis.

[CR130] Liu C-M, Ralph J, Fern L, Hermann T, Jenkins E, Liu M, Palleroni NJ, Prosser BL, Sello LH, Stempel A (1976). Studies on a new polyether antibiotic, Ro 21-6150. J Antibiot.

[CR131] Long P (1967). Studies on *Eimeria*
*praecox* Johnson, 1930, in the chicken. Parasitology.

[CR132] Long P (1968). The pathogenic effects of *Eimeria*
*praecox* and *E.*
*acervulina* in the chicken. Parasitology.

[CR133] Long PL, Millard BJ (1979). Immunological differences in *Eimeria* maxima: effect of a mixed immunizing inoculum on heterologous challenge. Parasitology.

[CR134] Long PL, Millard B, Joyner L, Norton CC (1976). A guide to laboratory techniques used in the study and diagnosis of avian coccidiosis. Folia Vet Lat.

[CR135] Luu L, Bettridge J, Christley RM, Melese K, Blake D, Dessie T, Wigley P, Desta TT, Hanotte O, Kaiser P (2013). Prevalence and molecular characterisation of *Eimeria* species in Ethiopian village chickens. BMC Vet Res.

[CR136] Marugan-Hernandez V, Jeremiah G, Aguiar-Martins K, Burrell A, Vaughan S, Xia D, Randle N, Tomley F (2020). The growth of *Eimeria*
*tenella*: characterization and application of quantitative methods to assess sporozoite invasion and endogenous development in cell culture. Front Cell Infect Microbiol.

[CR137] McDonald V, Rose ME (1987). *Eimeria*
*tenella* and *E.*
*necatrix*: a third generation of schizogony is an obligatory part of the developmental cycle. J parasitol.

[CR138] McDougald L, Jeffers T (1976). *Eimeria*
*tenella* (Sporozoa, Coccidia): gametogony following a single asexual generation. Science.

[CR139] McDougald LR, Fuller AL, McMurray BL (1990). An outbreak of Eimeria necatrix coccidiosis in breeder pullets: analysis of immediate and possible long-term effects on performance. Avian Dis.

[CR140] Mielke D (1993). A new system for the quantitative evaluation of the effectiveness of an *Eimeria*
*tenella* radiovaccine. Appl Parasitol.

[CR141] Mooney D, Richards K, Danaher M, Grant J, Gill L, Mellander P-E, Coxon C (2020). An investigation of anticoccidial veterinary drugs as emerging organic contaminants in groundwater. Sci Total Environ.

[CR142] Morgan JA, Godwin RM (2017). Mitochondrial genomes of Australian chicken *Eimeria* support the presence of ten species with low genetic diversity among strains. Vet Parasitol.

[CR143] Morris G, Gasser R (2006). Biotechnological advances in the diagnosis of avian coccidiosis and the analysis of genetic variation in *Eimeria*. Biotechnol Adv.

[CR144] Morris GM, Woods WG, Richards DG, Gasser RB (2007). Investigating a persistent coccidiosis problem on a commercial broiler–breeder farm utilising PCR-coupled capillary electrophoresis. Parasitol Res.

[CR145] Musa I, Sa’idu L, Jatau I, Adamu J, Otu M, Abdu P (2010). Outbreak of coccidiosis in 5-day old commercial broiler breeder flock in Zaria, Nigeria. Int J Poult Sci.

[CR146] Nematollahi A, Moghaddam G, Pourabad RF (2009). Prevalence of *Eimeria* species among broiler chicks in Tabriz (Northwest of Iran). Mun Ent Zool.

[CR147] Noack S, Chapman HD, Selzer PMJPR (2019). Anticoccidial drugs of the livestock industry. Parasitol Res.

[CR148] NOAH. NOAH compendium—datasheets. 2021. https://www.noahcompendium.co.uk/datasheets. Accessed 14 July 2021.

[CR149] Ogedengbe JD, Hunter DB, Barta JR (2011). Molecular identification of *Eimeria* species infecting market-age meat chickens in commercial flocks in Ontario. Vet Parasitol.

[CR150] Oikawa H, Kawaguchi H, Katagiri K, Nakamoto K (1979). Incidence of chicken coccidia from broiler houses in Japan, 1973–1977. Zentralblatt fur bakteriologie, parasitenkunde, infektionskrankheiten und hygiene. Erste abteilung originale. Reihe A Med Mikrobiol Parasitol.

[CR151] Osweiler G (2011). Ruminant toxicology, an issue of veterinary clinics: food animal practice—E-Book.

[CR152] Oviedo-Rondón E, Clemente-Hernández S, Williams P, Losa R (2005). Responses of coccidia-vaccinated broilers to essential oil blends supplementation up to forty-nine days of age. J Appl Poultry Res.

[CR153] Oviedo-Rondón E, Hume M, Hernández C, Clemente-Hernández S (2006). Intestinal microbial ecology of broilers vaccinated and challenged with mixed *Eimeria* species, and supplemented with essential oil blends. Poult Sci.

[CR154] Palafox A, Alicata J, Kartman L (1949). Breeding chickens for resistance to cecal coccidiosis1. Worlds Poult Sci J.

[CR155] Pastor-Fernández I, Kim S, Billington K, Bumstead J, Marugán-Hernández V, Küster T, Ferguson DJ, Vervelde L, Blake DP, Tomley FM (2018). Development of cross-protective *Eimeria*-vectored vaccines based on apical membrane antigens. Int J Parasitol.

[CR156] Peek H, Landman W (2011). Coccidiosis in poultry: anticoccidial products, vaccines and other prevention strategies. Vet Q.

[CR157] Pinard-Van Der Laan M, Monvoisin J, Pery P, Hamet N, Thomas M (1998). Comparison of outbred lines of chickens for resistance to experimental infection with coccidiosis (*Eimeria*
*tenella*). Poult Sci.

[CR158] Pinard-van der Laan M-H, Bed'Hom B, Coville J-L, Pitel F, Feve K, Leroux S, Legros H, Thomas A, Gourichon D, Repérant J-M (2009). Microsatellite mapping of QTLs affecting resistance to coccidiosis (*Eimeria*
*tenella*) in a Fayoumi×White Leghorn cross. BMC Genom.

[CR159] Quiroz-Castañeda RE, Dantán-González E (2015). Control of avian coccidiosis: future and present natural alternatives. BioMed Res Int.

[CR160] Reid WM (1990). History of avian medicine in the United States. X. Control of coccidiosis. Avian Dis.

[CR161] Reid AJ, Blake DP, Ansari HR, Billington K, Browne HP, Bryant J, Dunn M, Hung SS, Kawahara F, Miranda-Saavedra D, Malas TB, Mourier T, Naghra H, Nair M, Otto TD, Rawlings ND, Rivailler P, Sanchez-Flores A, Sanders M, Subramaniam C, Tay YL, Woo Y, Wu XK, Barrell B, Dear PH, Doerig C, Gruber A, Ivens AC, Parkinson J, Rajandream MA, Shirley MW, Wan KL, Berriman M, Tomley FM, Pain A (2014). Genomic analysis of the causative agents of coccidiosis in domestic chickens. Genome Res.

[CR162] Remnant JG (2007). Veterinary dictionary for students, 162.

[CR163] Ritzi MM, Abdelrahman W, Mohnl M, Dalloul RA (2014). Effects of probiotics and application methods on performance and response of broiler chickens to an *Eimeria* challenge. Poult Sci.

[CR164] Rose ME (1963). Some aspects of immunity to *Eimeria* infections. Ann NY Acad Sci.

[CR165] Rosenberg MM, Alicata JE, Palafox AL (1954). Further evidence of hereditary resistance and susceptibility to cecal coccidiosis in chickens. Poult Sci.

[CR166] Ryley JF, Wilson RG (1975). Laboratory studies with some recent anticoccidials. Parasitology.

[CR167] Salisch H (1989). Recent developments in the chemotherapy of parasitic infections of poultry. Worlds Poult Sci J.

[CR168] Scribner A, Dennis R, Hong J, Lee S, McIntyre D, Perrey D, Feng D, Fisher M, Wyvratt M, Leavitt P (2007). Synthesis and biological activity of imidazopyridine anticoccidial agents: part I. Eur J Med Chem.

[CR169] Scribner A, Dennis R, Lee S, Ouvry G, Perrey D, Fisher M, Wyvratt M, Leavitt P, Liberator P, Gurnett A (2008). Synthesis and biological activity of imidazopyridine anticoccidial agents: part II. Eur J Med Chem.

[CR170] Sharma S, Iqbal A, Azmi S, Mushtaq I, Wani ZA, Ahmad S (2015). Prevalence of poultry coccidiosis in Jammu region of Jammu and Kashmir State. J Parasit Dis.

[CR171] Shirley MW, Bedrník P (1997). Live attenuated vaccines against avian coccidiosis: success with precocious and egg-adapted lines of *Eimeria*. Parasitol Today.

[CR172] Shirley MW, Smith AL, Tomley FM (2005). The biology of avian *Eimeria* with an emphasis on their control by vaccination. Adv Parasitol.

[CR173] Soutter F, Werling D, Tomley FM, Blake DP (2020). Poultry coccidiosis: design and interpretation of vaccine studies. Front Vet Sci.

[CR174] Stayer P, Pote L, Keirs R (1995). A comparison of *Eimeria* oocysts isolated from litter and fecal samples from broiler houses at two farms with different management schemes during one growout. Poult Sci.

[CR175] Stephan B, Rommel M, Daugschies A, Haberkorn A (1997). Studies of resistance to anticoccidials in *Eimeria* field isolates and pure *Eimeria* strains. Vet Parasitol.

[CR176] Sun H, Wang L, Wang T, Zhang J, Liu Q, Chen P, Chen Z, Wang F, Li H, Xiao Y (2014). Display of *Eimeria*
*tenella* EtMic2 protein on the surface of *Saccharomyces*
*cerevisiae* as a potential oral vaccine against chicken coccidiosis. Vaccine.

[CR177] Swaggerty CL, Genovese KJ, He H, Duke SE, Pevzner IY, Kogut MH (2011). Broiler breeders with an efficient innate immune response are more resistant to *Eimeria*
*tenella*. Poult Sci.

[CR178] Takala SL, Plowe CV (2009). Genetic diversity and malaria vaccine design, testing and efficacy: preventing and overcoming ‘vaccine resistant malaria’. Parasite Immunol.

[CR179] Tang X, Liu X, Yin G, Suo J, Tao G, Zhang S, Suo X (2018). A novel vaccine delivery model of the apicomplexan *Eimeria*
*tenella* expressing *Eimeria* maxima antigen protects chickens against infection of the two parasites. Front Immunol.

[CR180] Tierney J, Gowing H, Van Sinderen D, Flynn S, Stanley L, McHardy N, Hallahan S, Mulcahy G (2004). In vitro inhibition of *Eimeria* tenella *invasion* by indigenous chicken *Lactobacillus* species. Vet Parasitol.

[CR181] ToxoDB. Organisms: genome info and stats. 2021. https://toxodb.org/toxo/app/search/organism/GenomeDataTypes/result?filterTerm=eimeria. Accessed 14 July 2021.

[CR182] Trees AJ, Jordan F, Pattison M, Alexander D, Faragher T (2001). Parasitic diseases. Poultry diseases.

[CR183] Vegad JL (2004). Poultry diseases: a guide for farmers and poultry professionals.

[CR184] Venkatas J, Adeleke M (2019). A review of *Eimeria* antigen identification for the development of novel anticoccidial vaccines. Parasitol Res.

[CR185] Vermeulen A (1998). Progress in recombinant vaccine development against coccidiosis a review and prospects into the next millennium. Int J Parasitol.

[CR186] Waldenstedt L, Elwinger K, Lunden A, Thebo P, Uggla A (2001). Sporulation of *Eimeria* maxima oocysts in litter with different moisture contents. Poult Sci.

[CR187] Walker RA, Ferguson DJ, Miller CM, Smith NC (2013). Sex and *Eimeria*: a molecular perspective. Parasitology.

[CR188] Wang C, Liu Y, Zheng H, Li Y, He J, Wang X, Wang M, Zhang L, Xue F, Zhang K (2020). Safety pharmacology assessment of ethanamizuril, a novel triazines coccidiostat. Res Vet Sci.

[CR189] Watkins KL, Brooks MA, Jeffers TK, Phelps PV, Ricks CA (1995). The effect of in ovo oocyst or sporocyst inoculation on response to subsequent coccidial challenge. Poult Sci.

[CR190] WHO (1965). World Health Organization technical report series no. 296.

[CR191] Williams R (1995). Epidemiological studies of coccidiosis in the domesticated fowl (*Gallus gallus*): II. Physical condition and survival of *Eimeria*
*acervulina* oocysts in poultry-house litter. Appl Parasitol.

[CR192] Williams R (1997). Laboratory tests of phenolic disinfectants as oocysticides against the chicken coccidium *Eimeria*
*tenella*. Vet Rec.

[CR193] Williams R (1998). Epidemiological aspects of the use of live anticoccidial vaccines for chickens. Int J Parasitol.

[CR194] Williams R (1999). A compartmentalised model for the estimation of the cost of coccidiosis to the world’s chicken production industry. Int J Parasitol.

[CR195] Williams R (2005). Intercurrent coccidiosis and necrotic enteritis of chickens: rational, integrated disease management by maintenance of gut integrity. Avian Pathol.

[CR196] Williams R, Marshall R, La Ragione R, Catchpole J (2003). A new method for the experimental production of necrotic enteritis and its use for studies on the relationships between necrotic enteritis, coccidiosis and anticoccidial vaccination of chickens. Parasitol Res.

[CR197] Williams R, Marshall R, Pages M, Dardi M, Del Cacho E (2009). Pathogenesis of *Eimeria*
*praecox* in chickens: virulence of field strains compared with laboratory strains of *E.*
*praecox* and *Eimeria*
*acervulina*. Avian Pathol.

[CR198] Zhang M, Li X, Zhao Q, She R, Xia S, Zhang K, Zhang L, Wang X, Wang M, Liu Y (2019). Anticoccidial activity of novel triazine compounds in broiler chickens. Vet Parasitol.

[CR199] Zhang K, Wang C, Li Y, He J, Wang M, Wang X, Zhang L, Fei C, Zheng H, Liu Y (2020). Rat two-generation reproductive toxicity and teratogenicity studies of a novel coccidiostat–ethanamizuril. Regul Toxicol Pharmacol.

[CR200] Zhang K, Wang X, Wang M, Liu Y, Zhang L, Wang C, Fei C, Li J, Xue F (2020). Rat 90-day oral toxicity study of a novel coccidiostat–ethanamizuril. Regul Toxicol Pharmacol.

[CR201] Zhu J, Lillehoj H, Allen P, Van Tassell C, Sonstegard T, Cheng H, Pollock D, Sadjadi M, Min W, Emara M (2003). Mapping quantitative trait loci associated with resistance to coccidiosis and growth. Poult Sci.

